# Mechanisms of Trained Innate Immunity in oxLDL Primed Human Coronary Smooth Muscle Cells

**DOI:** 10.3389/fimmu.2019.00013

**Published:** 2019-01-23

**Authors:** Lucia Schnack, Yahya Sohrabi, Sina M. M. Lagache, Florian Kahles, Dennis Bruemmer, Johannes Waltenberger, Hannes M. Findeisen

**Affiliations:** ^1^Department of Cardiology I, Coronary and Peripheral Vascular Disease, Heart Failure, University Hospital Münster, Münster, Germany; ^2^Department of Internal Medicine I-Cardiology, University Hospital Aachen, Aachen, Germany; ^3^Division of Cardiology, Department of Medicine, Pittsburgh Heart, Lung, Blood, and Vascular Medicine Institute, UPMC and University of Pittsburgh School of Medicine, Pittsburgh, PA, United States; ^4^Medical Faculty, University of Münster, Münster, Germany; ^5^Cells-in-Motion Cluster of Excellence (EXC 1003-CiM), University of Münster, Münster, Germany

**Keywords:** trained innate immunity, smooth muscle cells, inflammation, oxLDL, mTOR

## Abstract

**Objective:** Damage and pathogen associated molecular patterns such as oxidized low-density lipoprotein (oxLDL) or bacillus Calmette-Guerin (BCG) vaccine can induce long term pro-inflammatory priming in monocytes and macrophages due to metabolic and epigenetic reprogramming—an emerging new concept called trained innate immunity. Vascular smooth muscle cells express pattern recognition receptors involved in trained innate immunity in monocytes. Here we investigated whether the mechanisms of trained innate immunity also control a proinflammatory phenotype in human coronary smooth muscle cells.

**Methods:** Human coronary smooth muscle cells were primed with oxLDL or BCG for 24 h. After a resting time of 4 to 7 days, the cells were restimulated with either PAM3cys4, LPS or TNFα and cytokine production or mRNA expression were measured. Then, mechanisms of monocyte trained innate immunity were analyzed in smooth muscle cells, including receptors, intracellular pathways as well as metabolic and epigenetic reprogramming.

**Results:** Priming with oxLDL or BCG lead to a significantly increased production of IL6, IL8 and MCP-1 following restimulation. OxLDL priming had little effect on the expression of macrophage or SMC marker genes. Proinflammatory priming of smooth muscle cells induced mTOR-HIF1α-signaling and could be blocked by mTOR-, TLR2-, and TLR4-inhibition. Finally, metabolic and epigenetic mechanisms of trained innate immunity in monocytes could be replicated in smooth muscle cells, including increased glucose consumption, lactate production, responsiveness to 6-fluoromevalonate and mevalonate treatment and inhibition of priming by the histone methyltransferase inhibitor methylthioadenosine (MTA).

**Conclusion:** We demonstrate for the first time that mechanisms of the so called trained innate immunity control a proinflammatory phenotype in non-immune cells of the vascular wall. Our findings warrant further research into the specificity of trained innate immunity as an immune cell response as well as the mechanisms of vascular smooth muscle cells inflammation.

## Introduction

Cells of the innate immune system particularly monocytes and macrophages have been recognized as a major driving force of chronic vascular inflammation during initiation and progression of atherosclerosis. Pharmacologic modulation of this chronic inflammation is currently under investigation in large clinical trials as a treatment for atherosclerosis (1). The recent discovery of memory traits in cells of the innate immune system leading to chronic activation highlights an intriguing novel target for immunomodulatory treatment strategies (2). Previously, the innate immune system was considered to be an unspecific “first-line” of defense against infections, followed by a slower but more specific response of the adaptive immune system that also includes an immunologic memory for faster responses to future reinfection (3). However, cells of the innate immune system such as monocyte-derived and tissue-resident macrophages are characterized by a high phenotypic plasticity. This phenotypic plasticity facilitates a flexible response to immunologic challenges that can compromise tissue and organ function. Furthermore, depending on the trigger, these cells can acquire a more stable “memory-like” phenotype following specific priming. This “memory-like” phenotype allows a modulated response to an unrelated future challenge that can be enhanced, reduced or both (4). To describe a lasting activation that enables innate immune cells to develop an enhanced immune response to a secondary challenge, Netea et al. have coined the term “trained innate immunity” (5). They have conclusively demonstrated that trained innate immunity in response to infection or vaccination represents an immunologic mechanism protecting against future infections that are not necessarily related to the initial trigger (5). Mechanistically, trained innate immunity provides a sustained activation to restimulation leading to an enhanced and specific transcriptional response (6). Exposure of isolated human monocytes to the bacillus Calmette-Guerin (BCG) vaccine or cell wall components of candida albicans (β-glucan) induces a persistent inflammatory activation with increased synthesis of chemo- and cytokines upon toll-like receptor (TLR) stimulation after 6 days. A similar response could be observed in monocytes isolated from healthy human subjects 3 months after BCG vaccination (3, 7). Current evidence has consistently implicated metabolic and epigenetic mechanisms in the regulation of this trained innate immunity (8, 9). Priming of monocytes by β-glucan stimulation was associated with a shift from oxidative phosphorylation to glycolysis resulting in increased lactate production in an Akt/mTOR/Hif1α-dependent manner (6, 8). Furthermore, activation of glutaminolysis and cholesterol synthesis has been demonstrated to control trained immunity (8, 9). Downstream, metabolic reprogramming controls modulation of the epigenetic landscape. Pharmacologic inhibition of histone methyltransferases or of the described metabolic pathways can block the emergence of trained monocytes (8, 10). While the trained innate immunity phenotype can confer protection from infectious diseases, it can also be induced by non-microbial triggers. Preliminary studies demonstrating the induction of a trained phenotype in response to atherogenic stimuli such as oxidized low-density lipoprotein (oxLDL) suggest a role in sterile chronic inflammatory diseases including atherosclerosis (2, 11). *In vitro*, exposure of human monocytes for 24 h to atherogenic particles such as oxLDL results in increased expression of the inflammatory mediators TNFα, IL6, MCP-1, and MMP-9 upon restimulation with TLR2 and 4 agonists after 6 days as well as increased foam cell formation. Induced cytokines and chemokines revealed H3K4me3 enrichment on their promoter regions, while inhibition of histone methyltransferases blocked the emergence of inflammatory primed cells (11). Furthermore, circulating monocytes of patients with symptomatic atherosclerosis had a pro-inflammatory phenotype and increased expression of glycolytic enzymes, associated with epigenetic remodeling (12).

While cells of the immune system, particularly monocytes and macrophages, have been at the center of research focusing on vascular inflammation, the importance of smooth muscle cells (SMCs) in this context has increasingly been recognized. Rigorous lineage tracking experiments have recently demonstrated that significant numbers of macrophage-marker expressing cells in atherosclerotic lesions are in fact of smooth muscle origin (13–15). Furthermore, vascular SMCs are capable of expressing toll-like receptors and other pattern recognition receptors that can recognize oxLDL as damage-associated molecular pattern (DAMP) (16, 17). Therefore, we investigated if the mechanisms of trained innate immunity also control a proinflammatory phenotype in human coronary smooth muscle cells.

## Materials and Methods

### Cell Culture

Different batches of human coronary smooth muscle cells were commercially obtained from ThermoFisher. For all experiments cells were grown to 80% confluence in Growth Medium (231M ThermoFisher + SMGM) and then serum-deprived for 48 h in Basal Medium (231M ThermoFisher) with 0.4% FBS. Cells were pretreated with OSI-027 (Cayman 17379) Torin 1 (Cayman 10997), MTA (Biomol, Cay15593-25), CU-CPT22 (Calbiochem Millipore 614305), TAK-242 (InvivoGen, tlr1-cli95), Mevalonate (SIGMA, 50838) or 6-Fluormevalonate (SIGMA, F2929) for 30 min, then primed with oxLDL (5 to 20 μg/ml), native LDL (nLDL) (10 to 20 μg/ml) or BCG (2.5 to 5 μg/ml) for 24 h. Cells were washed once and incubated in medium. After 3 to 6 days of additional incubation in Basal Medium + 0.4% FBS, cells were exposed to either medium alone, 50 ng/ml LPS (SIGMA, L6529), 10 ng/μl TNFα (Reliatech, 300-027) or 5 μg/ml PAM3Cys4 (EMC mirocollection, L2000) for up to 24 h. Lyophilized BCG vaccine (Strain Pasteur 1173P2) was kindly provided by the Pasteur Institute of Iran, dissolved in H2O and prior to use bacteria were inactivated by multiple freeze-thaw cycles.

### LDL-Isolation

LDL was isolated from blood plasma of healthy donors as described by Van Tits et al. (16). Briefly, the LDL-Fraction was separated by two 24 h steps of ultracentrifugation at 4°C. After this, 4 dialysis steps against PBS (pH 7.4, 4°C) were performed in increasing time intervals [(1) 1 h, (2) 2 h, (3) 3 h, (4) overnight]. The obtained nLDL was sterile filtered and oxidized with 20 μM CuSO4 for 24 h at 37°C. After oxidation another 4 cycles of dialysis against PBS followed, as described above. OxLDL was sterile filtered and protein concentration was measured with a Lowry Protein Assay (ThermoFisher, 23240) following the manufacturer's instructions. TBARS were measured with OxiSelect TBARS Assay Kit (Cell Biolabs, 1024311). Experiments in the manuscript were performed with low TBAR LDL. To detect possible endotoxin contamination an endotoxin assay was performed with a commercially obtained kit (GenScript, ABIN491527). All nLDL or oxLDL which was used in the experiments had endotoxin levels lower than 0.01 EU/ml.

### ELISA

For ELISA supernatant of the cells was collected 24 h after restimulation and stored until usage at −20°C. The assay was performed using R&D DuoSet ELISA Development System (Human IL6 DuoSet ELISA, #DY206; Human IL8/CX-CL8 DuoSet ELISA #DY208; Human CCL2/MCP-1 DuoSet ELISA #DY279), following the manufacturer's instructions. Absorbance was measured using a VICTOR X3 multimode Plate Reader at 450 nm, concentrations were calculated by four parameters logistic regression.

### Real-Time Quantitative PCR

For real-time qPCR, cells were primed as described above and restimulated on day 5 for 6 h. RNA was isolated using a spin-column based RNA isolation kit (Machery-Nagel, 1015722) according to the manufacturer's instruction. RNA concentration was measured using NanoDrop, isolated RNA was then reverse-transcribed using the RevertAid First Strand cDNA Synthesis Kit (ThermoFisher, K1632). Real-time qPCR was performed using the SYBR Green method (Bio-Rad, 102393). TFIIb was used as a housekeeping gene. Primer sequences can be found in the supplement.

### Western Blotting

Expression of p70S6K, phospho-p70S6K, NFκB-p65 and phospho-NFκB-p65 in primed smooth muscle cells were measured in cell lysate by western blot. Cells were primed with oxLDL as described above and lysed after 4 h. To analyze expression of Hif1α, cells were primed with oxLDL as described above and harvested on day 5. 7.5 to 10% acrylamide gels were loaded with equal amount of protein per lane. Proteins were blotted onto a PVDF membrane and blocked in 5% milk (w/v) in Tris-buffered saline supplemented with Tween 20 (TBS-T). Membranes were incubated in primary antibody [p70S6 Kinase (49D7) Rabbit Antibody, Cell Sigaling, 2708; Phospho-p70S6 Kinase (Thr389) Antibody, Invitrogen 710095; phospho-NFκB p65 (Ser536) Antibody, Cell Signaling 3033; NFκB p65 (F-6), Santa Cruz, Sc-8008; Hif-1α Antibody, BD 610959; Vinculin Antibody (7F9), Santa Cruz, Sc-73614] in TBS-T overnight at 4°C, washed 3 times and incubated in secondary antibody (Goat anti-rb IgG- HRP, Santa Cruz, sc-2004; Goat anti-mouse IgG-HRP Santa Cruz, sc-2005) in TBS-T for 1 h. For development of the membranes Pierce ECL Western Blotting Substrate (ThermoFisher, 32106) was used.

### Apoptosis Assay

Cells were treated with oxLDL as mentioned above. On day 5 apoptosis was analyzed by using the TiterTACS *in situ* Detection Kit (R&B) according to the manufacturer's protocol. Briefly, cells were fixed in 3.7% buffered formaldehyde and post-fixed in 100% methanol. A positive control was created by treating the cells with TACS-Nuclease™ to generate DNA breaks in every cell. Apoptotic cells were measured, using a VICTOR X3 multimode Plate Reader at 450 nm.

### Lactate Assay

Intracellular Lactate was measured by using a colorimetric L-Lactate assay kit according to the manufactures instruction (abcam, ab65330). Cells were treated with oxLDL for 24 h and lysed on day 5. To eliminate endogenous LDH, cell lysate was deproteinized by spinning through a 10kD Spin column (ab93349). Absorbance was measured with a CLARIOstar Microplate Reader at 570 nm.

### Glucose Consumption Assay

To measure glucose consumption, cells were treated with oxLDL as described above. On day 5 cells were washed once and fresh medium was applied. After 24 h glucose concentration was measured in the supernatant with a colorimetric glucose assay kit by abcam (ab65333), following the manufactures instructions. Absorbance was measured with a CLARIOstar Microplate Reader at 570 nm.

### Proliferation Assay

Cells were treated with the indicated doses of Torin 1, OSI-027, CU-CPT22, TAK-242, or MTA for 24 h, cells were washed once and incubated in medium. After 3 days of additional incubation in Basal Medium + 0.4% FBS, cells were washed once again and growth factor supplemented Medium was applied, to stimulate cell proliferation. After 24 h cells were detached with Trypsin and counted with FACS (Guava easyCyte, Millipore).

### Statistics

Each experiment was performed at least 3 times. All data were expressed as arithmetic mean + SEM. Statistical analysis was performed with an unpaired Student‘s *t*-test or one-way ANOVA. Statistical significance was defined as *P* < 0.05.

## Results

### oxLDL Induces a Dose-Dependent Proinflammatory Priming Effect in SMCs

**Figure 1:**
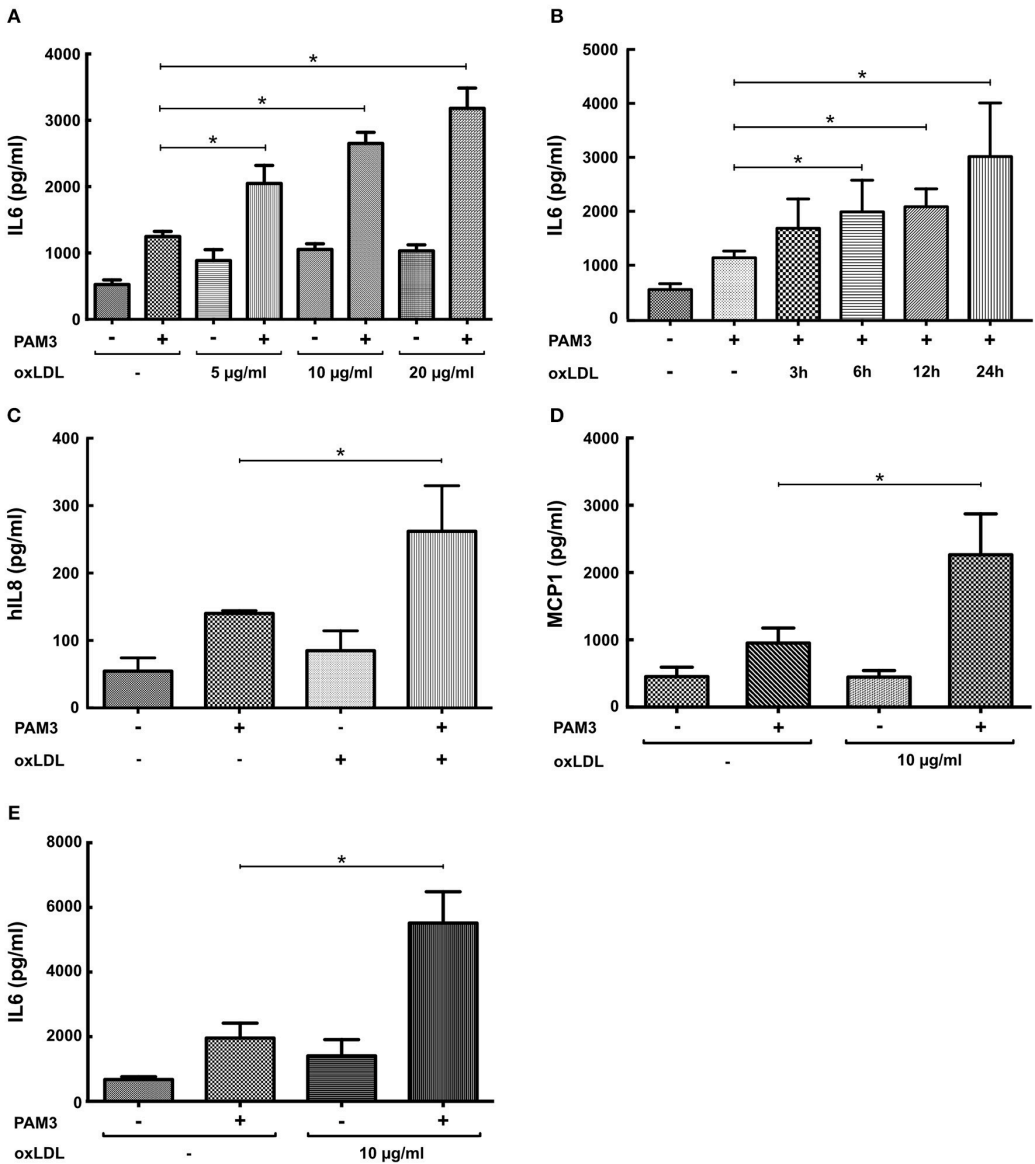
OxLDL induces a dose-dependent proinflammatory priming effect in SMCs. **(A)** SMCs were treated with different doses of oxLDL for 24 h, on day 5 cells were restimulated with 5 μg/ml PAM3cys4 for 24 h and IL6 levels were analyzed in the supernatant. **(B)** SMCs were treated with 10 μg/ml oxLDL for 3 to 24 h and restimulated with 5 μg/ml PAM3cys4 on day 5. IL6 levels were analyzed in the supernatant. **(C,D)** SMCs were treated with 10 μg/ml oxLDL for 24 h. On day 5 cells were restimulated with 5 μg/ml PAM3cys4 for 24 h and IL8 levels **(C)** and MCP-1 levels **(D)** were analyzed in the supernatant. **(E)** SMCs were treated with 10 μg/ml oxLDL for 24 h and restimulated with 5 μg/ml PAM3cys4 on day 8 (^*^p < 0.05, SEM, all experiments were repeated at least 3 times).

**Figure 2:**
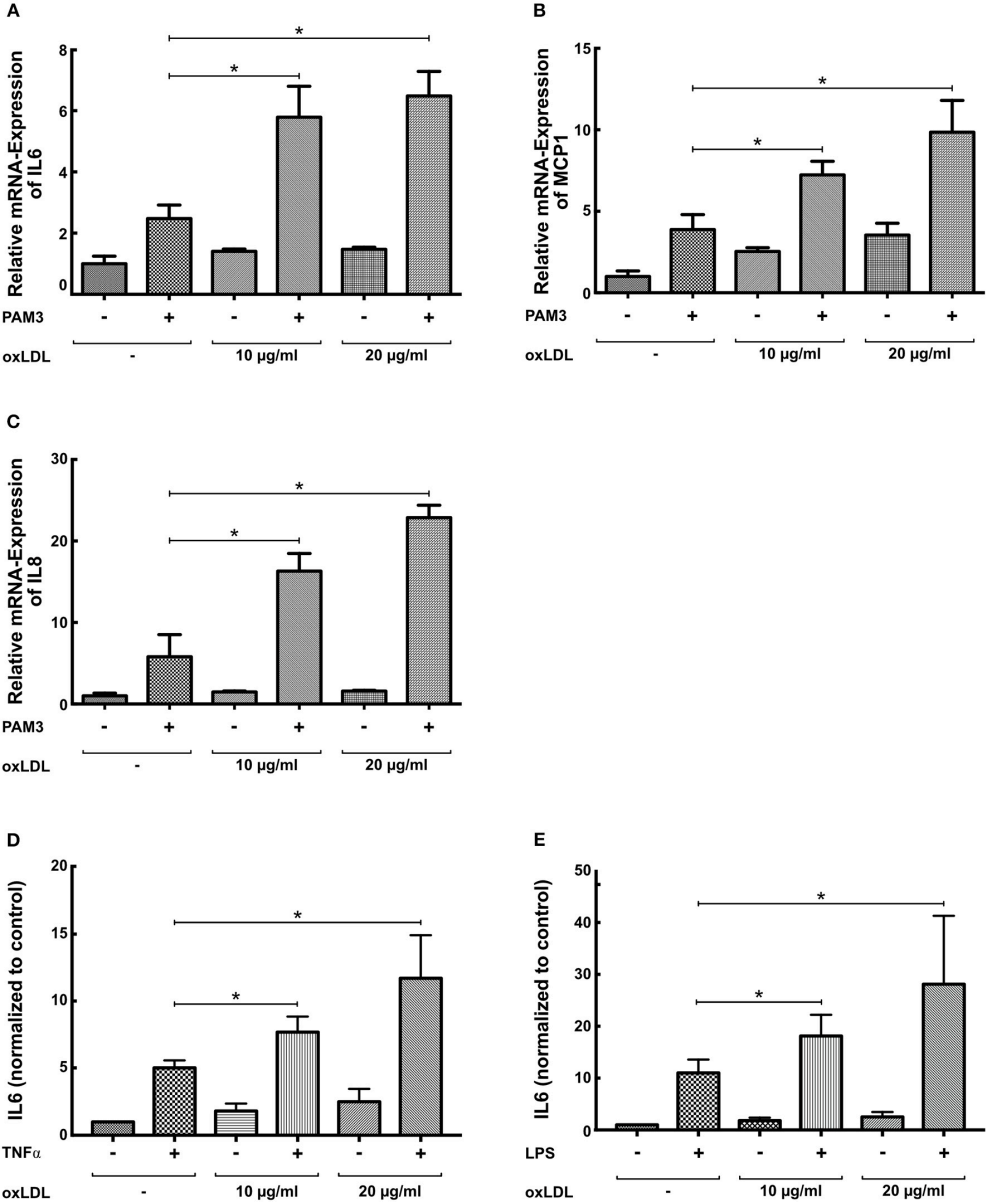
OxLDL priming induces mRNA levels of inflammatory cytokines. **(A–C)** SMCs were treated with 10 μg/ml oxLDL for 24 h, and restimulated with 5 μg/ml PAM3cys4 for 6 h on day 5. mRNA levels were analyzed by real-time qPCR. **(D,E)** SMCs were treated with the indicated doses of oxLDL for 24 h and restimulated with 10 ng/ml TNFα **(D)** or 50 ng/ml LPS for 24 h. IL6 levels were analyzed in the supernatant (^*^p < 0.05, SEM, all experiments were repeated at least 3 times).

First, replicating the experiments of Bekkering et al. in human monocytes (11), we utilized a similar experimental setup to study oxLDL priming in human coronary SMCs. SMCs were primed with oxLDL for 24 h and rested for 3 days. On day 5, cells were restimulated with the TLR2-agonist PAM3cys4 resulting in a dose-dependent increase in IL6 production while priming with nLDL had no effect (Figure 1A, Figure S1). As depicted in Figure 1B, duration of oxLDL incubation also influenced IL6 levels upon restimulation. OxLDL priming also increased levels of other important inflammatory cytokines including IL8 and MCP-1 (Figures 1C,D, Figure S2). In human monocytes an enhanced inflammatory response was documented up to 6 days after oxLDL priming (11). In SMCs we could demonstrate a strong induction of IL6 after restimulation for up to 7 days (Figure 1E). OxLDL priming resulted in sustained proinflammatory priming without inducing significantly increased apoptosis or necrosis (Figure S3). OxLDL priming also induced mRNA levels of IL6, IL8, and MCP-1 upon restimulation suggesting transcriptional regulation (Figures 2A–C). As depicted in Figures 2D,E restimulation with TNFα or LPS could also induce an enhanced inflammatory response. However, as we observed the strongest and consistent response with the TLR2-Agonist PAM3cys4 and considering the importance of vascular wall TLR2 in atherosclerosis formation (18), we performed subsequent experiments using the TLR2-agonist PAM3cys4. Together, these experiments demonstrate a proinflammatory response of SMCs to oxLDL-priming.

### BCG Priming Induces a Proinflammatory Priming Effect in SMCs

**Figure 3:**
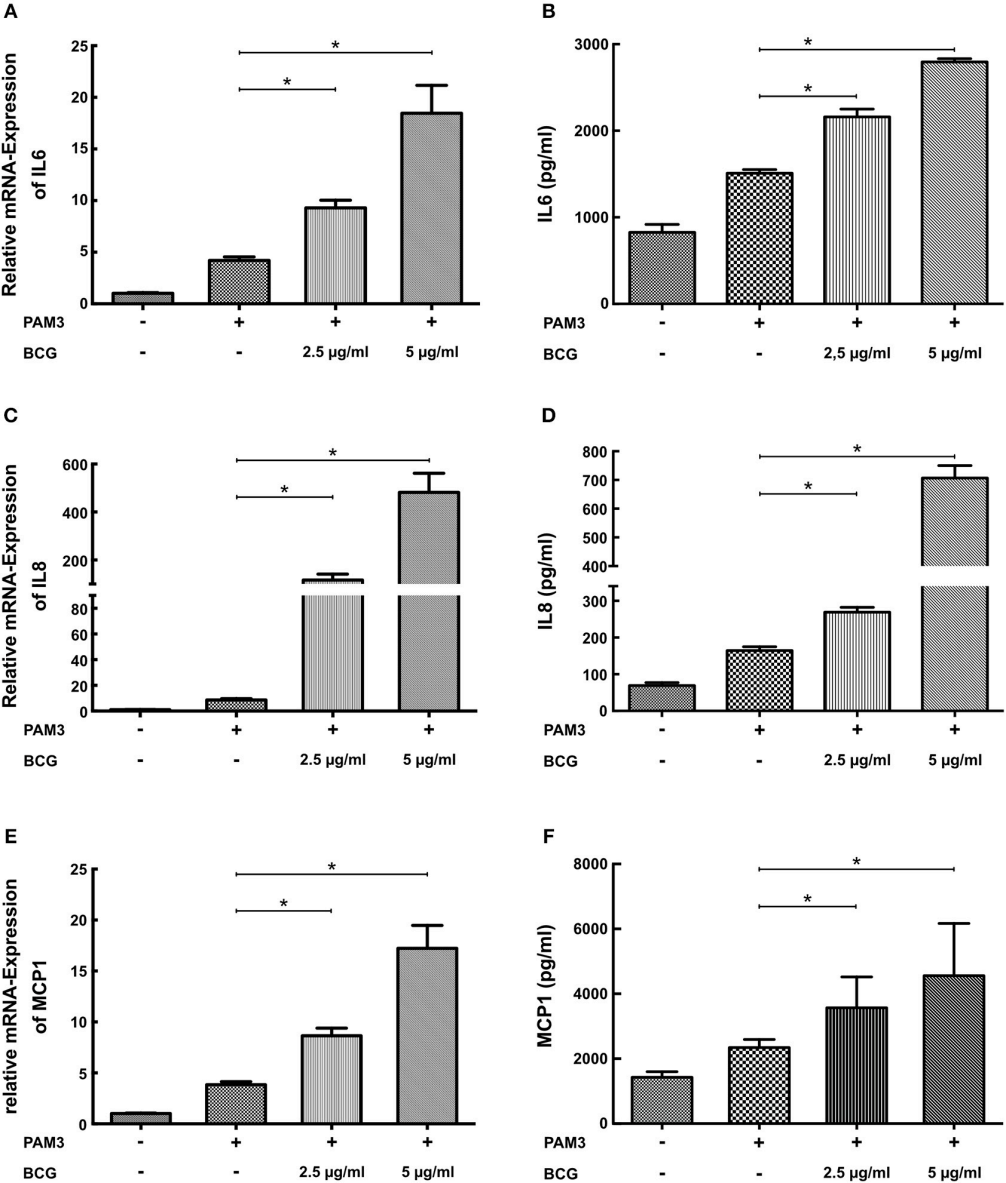
BCG induces a dose-dependent proinflammatory priming effect in SMCs. **(A,C,E)** SMCs were treated with the indicated doses of BCG for 24 h and restimulated with 5 μg/ml PAM3cys4 for 6 h on day 5. mRNA levels of Il6, IL8, and MCP1 were analyzed by real-time qPCR. **(B,D,F)** SMCs were treated with the indicated doses doses of BCG for 24 h, on day 5 cells were restimulated with 5 μg/ml PAM3cys4 for 24 h and IL6 levels **(B)**, IL8 levels **(D)**, and MCP-1 levels **(F)** were analyzed in the supernatant (^*^p < 0.05, SEM, all experiments were repeated at least 3 times).

Macrophage trained innate immunity was first described in response to pathogen associated molecular patterns (PAMPs) such as BCG or β-glucan (5, 6). Therefore, we have analyzed the effect of BCG priming on SMCs. As demonstrated in Figure 3 BCG priming was also able to induce a proinflammatory response to PAM3cys4 restimulation with a strong induction of mRNA and protein levels of IL6, IL8, and MCP-1.

### OxLDL Priming Has Little Effect on the Expression of Macrophage and SMC Marker Genes

**Figure 4:**
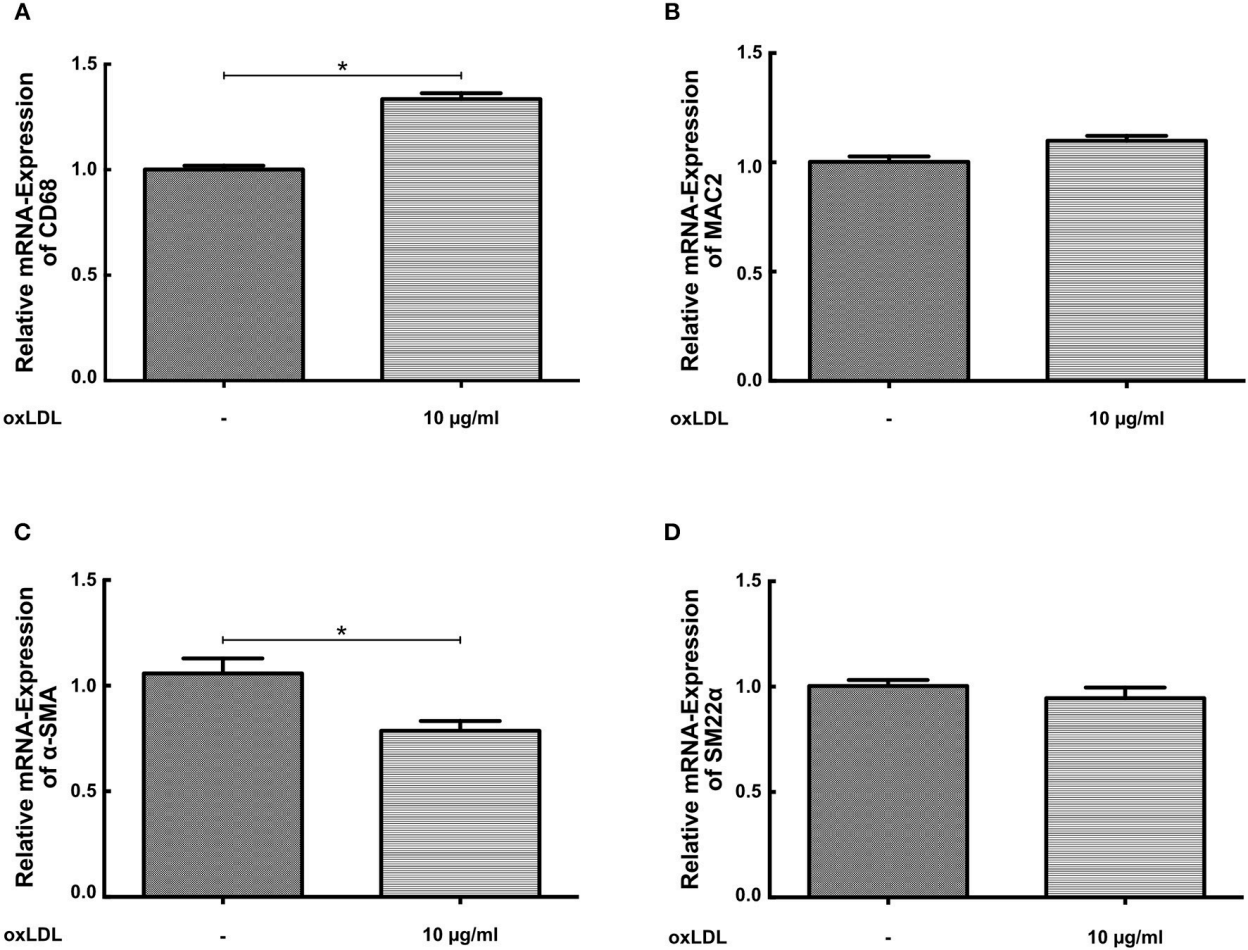
OxLDL priming has little effect on the expression of macrophage and SMC marker genes. **(A–D)** SMCs were treated with 10 μg/ml oxLDL for 24 h. mRNA levels were analyzed by real-time qPCR (^*^p < 0.05, SEM, all experiments were repeated at least 3 times).

SMCs display a considerable phenotypic variability in response to different environmental cues. During atherosclerosis formation SMCs can start to express macrophage markers with concomitant down regulation of traditional SMC markers (19, 20). Therefore, we analyzed the expression of macrophage and SMC markers in response to oxLDL priming. As illustrated in Figure 4 oxLDL priming somewhat increased the expression of the macrophage marker CD68 but had no effect on the expression of the marker MAC2 (Figure 4B). Conversely, expression of α-SMA was reduced (Figure 4C). However, expression of the more stable SMC marker SM22α was virtually unchanged (Figure 4D).

### Inhibition of TLR2- and TLR4-Signaling as Well as mTOR-Signaling Blocks oxLDL Priming

**Figure 5:**
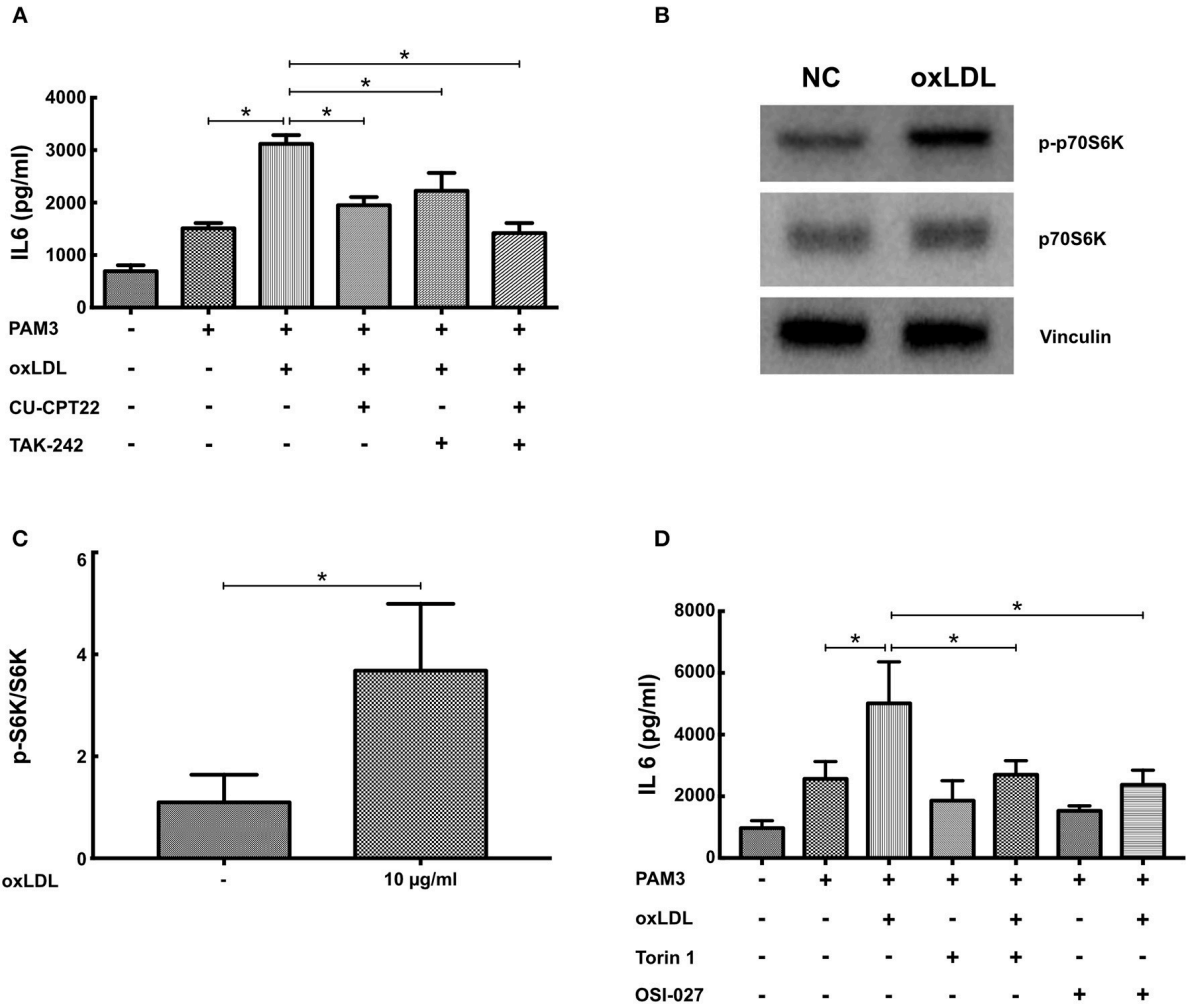
Inhibition of TLR2-, TLR4-, and mTOR-signaling blocks oxLDL priming. **(A)** SMCs were pretreated with 5 μM of the TLR2-inhibitor CU-CPT22 or 1 μM of the TLR4-inhibitor TAK242 or both for 30 min, followed by 10 μg/ml oxLDL for 24 h. Cells were restimulated on day 5 with 5 μg/ml PAM3 for 24 h and IL6 levels were analyzed in the supernatant. **(B,C)** SMCs were treated with 10 μg/ml oxLDL for 4 h. Cell lysates were analyzed by western blot. **(D)** SMCs were pretreated with 10 nM of the mTOR-inhibitor Torin1 or 1 μM of the mTOR-inhibitor OSI27 for 30 min, followed by 10 μg/ml oxLDL for 24 h. Cells were restimulated on day 5 with 5 μg/ml PAM3cys4 for 24 h and IL6 levels were analyzed in the supernatant (^*^p < 0.05, SEM, all experiments were repeated at least 3 times).

Training of human monocytes depends on TLR- and mTOR-signaling (6, 11). Therefore, we analyzed the relevance of both signaling pathways to oxLDL priming in SMCs. As depicted in Figure S4 oxLDL priming induced the activation of NFκB, a major downstream target of TLR-signaling. Similar to human monocytes, pharmacologic inhibition of TLR2 (CU-CPT22) and TLR4 (TAK242) could attenuate oxLDL priming. The simultaneous inhibition of both receptors could almost completely block the primed phenotype (Figure 5A). mTOR signaling was also activated by oxLDL priming as shown by increased phosphorylation of p70S6K (Figures 5B,C). Furthermore, pharmacologic inhibition of mTOR using two different mTOR-inhibitors (Torin1 and OSI-027) could block the emergence of the primed phenotype (Figure 5D, Figure S5). To rule out toxic or non-specific effects of the inhibitors used in these experiments, we analyzed proliferation following inhibitor treatment. There was no effect of inhibitor treatment on proliferation (Figure S6). Together, these experiments demonstrate the involvement of identical intracellular pathways in monocytes and SMCs in response to oxLDL priming.

### Metabolic and Epigenetic Regulation of oxLDL Priming in SMCs

**Figure 6:**
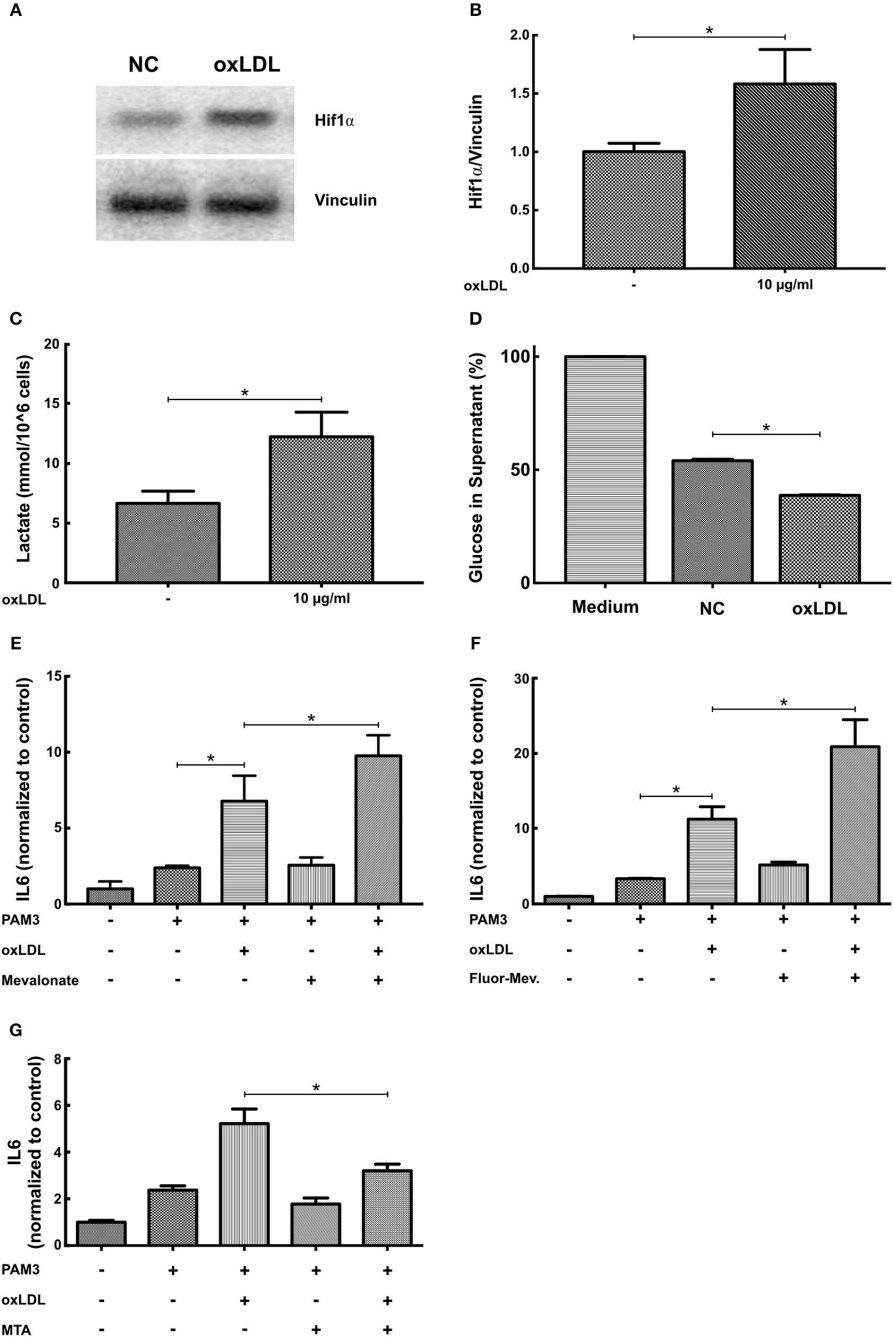
OxLDL priming involves metabolic and epigenetic mechanisms. **(A,B)** SMCs were treated with 10 μg/ml oxLDL for 24 h. After 3 days resting time cells were harvested and HIF1α protein levels were analyzed by western blot. **(C)** SMCs were treated with 10 μg/ml oxLDL for 24 h. After 3 days resting time cells were lysed and lactate concentration measured. **(D)** SMCs were treated with 10 μg/ml oxLDL for 24 h. After 3 days resting time fresh medium was applied and after another 24 h glucose concentration was measured in the supernatant. **(E–G)**: SMCs were pretreated with 3 mM mevalonate **(E)** or 1 mM 6-fluoromevalonate **(F)** or 10 μM of the histone methyltransferase inhibitor MTA **(G)** for 30 min, followed by 10 μg/ml oxLDL for 24 h. Cells were restimulated on day 5 with 5 μg/ml PAM3cys4 for 24 h and IL6 levels were analyzed in the supernatant (^*^p < 0.05, SEM, all experiments were repeated at least 3 times).

BCG and oxLDL trained innate immunity in monocytes is associated with a profound metabolic reprogramming. Activation of the mTOR-HIF1α-axis during training leads to increased glycolysis as well as pyruvate and lactate production (6, 10). Increased glycolysis and pyruvate levels can activate cholesterol synthesis leading to accumulation of the metabolite mevalonate. Mevalonate itself was shown to be a mediator of training in human monocytes (10). Again replicating the monocyte phenotype, oxLDL priming of SMCs increased HIF1α protein levels (Figures 6A,B), expression of HIF1α target genes LDH and HK2 (Figure S7), lactate production and glucose consumption, indicating increased glycolysis (Figures 6C,D) (10). In contrast to the observations of Bekkering et al. in monocytes, adding 6-fluoromevalonate or mevalonate instead of oxLDL did not induce priming in SMCs. However, mevalonate as well as 6-fluoromevalonate could strongly superinduce oxLDL priming (Figures 6E,F). Finally, as oxLDL training of human monocytes could be blocked by the histone methyltransferase inhibitor methylthioadenosine (MTA), we investigated the effects of MTA on oxLDL priming in SMCs. As illustrated in Figure 6G, MTA could block proinflammatory oxLDL priming in SMCs as well.

## Discussion

Priming or training of innate immune cells has been implicated to contribute to chronic vascular inflammation, a fundamental mechanism of atherosclerosis formation. In the present study, we report a previously unrecognized mechanism of sustained SMC inflammation replicating established cellular mechanisms of trained innate immunity in non-immune cells. Priming of human coronary SMCs with oxLDL or BCG can poise the cells to an increased synthesis of chemokines and cytokines upon restimulation. OxLDL priming involves mTOR- and TLR-dependent signaling pathways as well as metabolic and epigenetic regulatory mechanisms.

Differentiated SMCs resident in the normal vessel display a contractile phenotype enabling them to regulate vascular tone and diameter. However, similar to macrophages, SMCs retain an impressive phenotypic plasticity in response to certain environmental cues (21, 22). Activated SMCs downregulate the expression of contractile genes and potentiate their ability to migrate, proliferate, and secrete extracellular matrix proteins or inflammatory mediators, depending on the initial stimulus. The phenotypic plasticity of SMCs is increasingly recognized as a pivotal component of vascular remodeling and atherosclerosis formation (21, 22). Previous research has demonstrated that an activated SMC phenotype can be induced through growth factors or inflammatory mediators (14) but also through PAMPs and DAMPs such as chlamydial Heat Shock Protein 60 or oxLDL (23, 24). TLRs are crucial mediators of this phenotypic switch in response to PAMPs and DAMPs. Furthermore, although a role of infections in the pathogenesis of atherosclerosis has never been firmly established (25), SMCs can be direct targets of infectious particles such as human cytomegalovirus or *Chlamydia pneumoniae* (24, 26, 27). Here, we extend these observations and report that DAMPs and PAMPs such as oxLDL and BCG can induce a sustained activation of SMCs with enhanced secretion of inflammatory cytokines in response to restimulation. Furthermore, we demonstrate that primed SMCs exhibit mechanistic features known to regulate monocyte or macrophage trained innate immunity such as activation of the mTOR-HIF1α-axis and subsequent induction of aerobic glycolysis and responsiveness to metabolites of the mevalonate pathway (6, 10). So far, metabolic adaptions during SMC phenotypic switch have been addressed only by a few studies in the context of growth factor stimulation (28). Lambert et al., Perez et al., and Xiao et al. have analyzed the metabolic consequences of PDGF stimulation of SMC. Similar to our findings they demonstrated the activation of HIF1α, enhanced aerobic glycolysis and increased expression of HK2 and LDH (29–31). Together with our data these studies suggest that activation of the mTOR-HIF1α-axis and increased glycolysis are common features of SMC activation by growth factors and DAMPs. Similar to monocytes or macrophages, activated SMCs have an increased demand of glucose and metabolic intermediates to carry out their designated function (28). Further research is necessary to elucidate the detailed metabolic processes and investigate possible links to the epigenetic machinery that has been described in macrophages. While trained innate immunity is an adaptive response of the innate immune systems of a host organism to an infectious stimulus, conferring increased protection to recurrent infections (5), the functional role of the observed SMC response to PAMPs remains elusive. However, considering the recent findings by Naik et al. that epithelial stem cells can develop a memory of previous inflammatory insults facilitating faster wound healing, it appears conceivable that the observed phenotypes are part of a more global but unspecific response to cellular stressors to adapt the organism to expected future challenges (32).

Furthermore, rigorous lineage tracking experiments have conclusively demonstrated that during atherosclerosis formation medial smooth muscles cell undergo phenotypic transitions and activate markers of several different cell types including macrophages. A significant number of macrophage-like cells in murine atherosclerotic lesions are in fact of SMC origin. These SMC-derived macrophages contribute significantly to the formation of advanced atherosclerotic lesions (19, 20). *In vitro*, cholesterol loading of SMCs also results in the transition of SMCs to macrophage-like cells that show enhanced phagocytic activity with foam cell formation and increased expression of inflammatory genes (19, 33). It appears conceivable that during phenotypic transition SMC derived macrophage-like cells activate metabolic and epigenetic programs known to control immunologic functions in macrophages.

Recent *in vivo* studies have demonstrated that modulation of myeloid progenitors in the bone marrow by DAMPs and PAMPs is an integral component of trained innate immunity (34–36). Therefore, priming of progenitor cells appears to be the key to long-term phenotypic modulation. This is of significant relevance to SMCs as additional studies have demonstrated that SMC-derived cells in atherosclerotic lesions originate from a small subset of medial SMCs that undergo clonal expansion suggesting the presence of progenitor cells in the media (15, 37). Finally, adult heterozygous apolipoprotein E (apoE^+/−^) offspring from hypercholesterolemic apoE^−/−^ mothers showed significant changes in histone methylation patterns in vascular smooth muscle cells as compared with genetically identical apoE^+/−^ offspring from normocholesterolemic wild-type mothers, demonstrating sustained epigenetic changes *in vivo* in response to a proatherogenic environment (38). Therefore, if DAMPs and PAMPs are able to train SMC-progenitor cells toward a proinflammatory phenotype, this could have long-lasting and substantial effects on vascular inflammation and atherosclerosis formation. Additional studies are necessary to elucidate the presence and a potential role of DAMP or PAMP induced priming on smooth muscle cells *in vivo*.

In conclusion, we demonstrate for the first time that the mechanisms of the so called trained innate immunity in response to oxLDL and BCG control a proinflammatory phenotype in non-immune cells of the vascular wall. Collectively, these experiments warrant further research into the specificity of trained innate immunity as an immune cell response as well as the mechanisms of vascular smooth muscle cell inflammation.

## Author Contributions

LS, YS, and SL performed experiments. FK and DB assisted in designing and performing experiments. JW designed experiments, assisted in drafting the manuscript. HF designed the study, drafted the manuscript.

### Conflict of Interest Statement

The authors declare that the research was conducted in the absence of any commercial or financial relationships that could be construed as a potential conflict of interest.
