# Supplementary material for: Mechanisms of Trained Innate Immunity in oxLDL Primed Human Coronary Smooth Muscle Cells
Source: Front Immunol. 2019 Jan 23;10:13. doi: 10.3389/fimmu.2019.00013 (PMC6351498; doi:10.3389/fimmu.2019.00013)
Supplement: Supplementary file 1 [file Data_Sheet_1.pdf]

Supplementary Material

Mechanisms of Trained Innate Immunity in oxLDL Primed Human Coronary Smooth Muscle Cells

Lucia Schnack, Yahya Sohrabi, Sina M. M. Lagache, Florian Kahles, Dennis Bruemmer, Johannes Waltenberger, Hannes M. Findeisen

Correspondence: Hannes M. Findeisen: hannes.findeisen@ukmuenster.de

Supplementary Figures

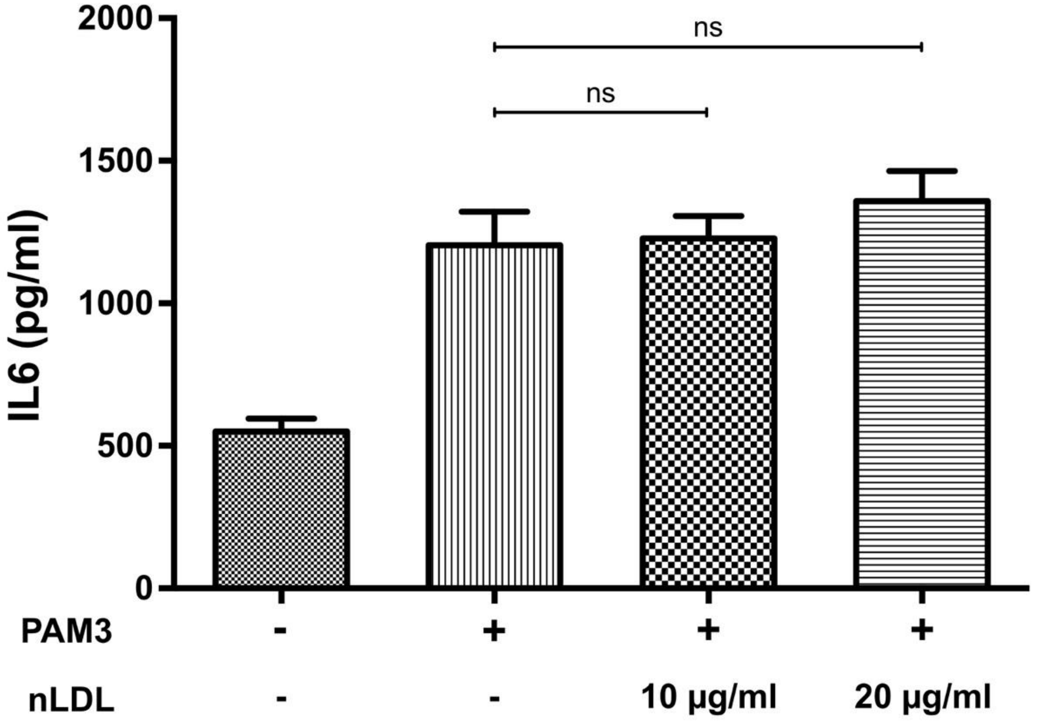

Figure S1

nLDL treatment does not induce priming in SMCs. Cells were treated 10 or 20 µg/ml nLDL for 24h. On day 5 cells were restimulated with 5 µg/ml PAM3cys4 for 24h and IL6 levels were analyzed in the supernatant. (* $p < 0.05$, SEM, all experiments were repeated at least 3 times).

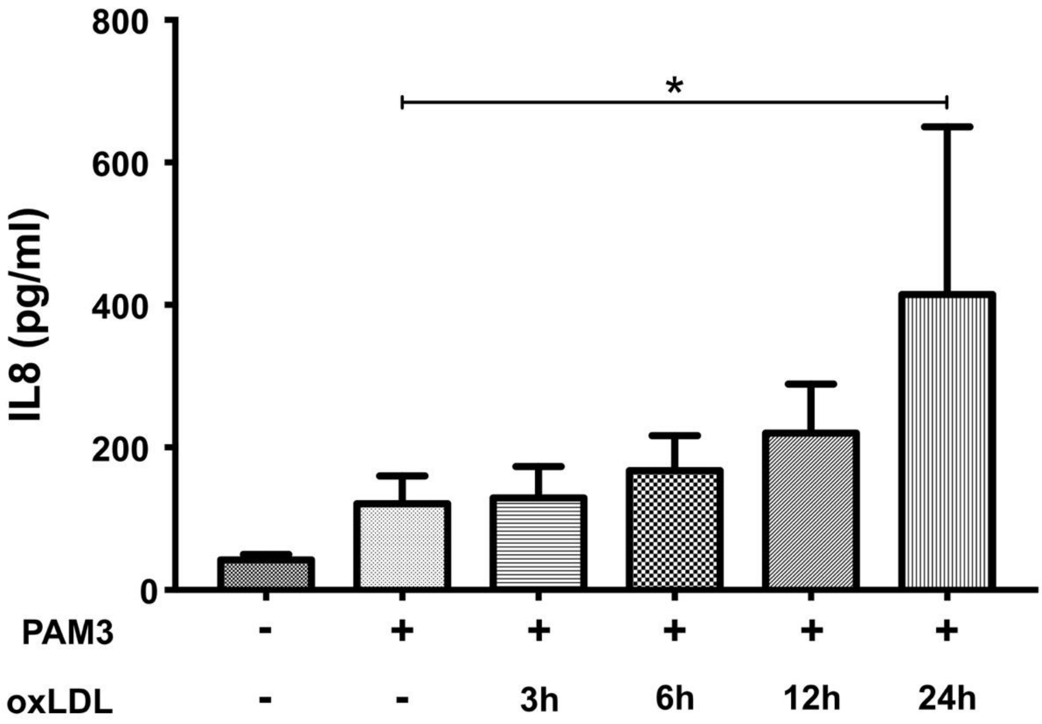

Figure S2

Levels of IL8 secretion upon restimulation in oxLDL-treated SMCs depend on the duration of priming. Cells were treated with 10 µg/ml oxLDL for 3 up to 24h. On day 5 cells were restimulated with 5 µg/ml PAM3cys4 for 24h and IL8 levels were analyzed in the supernatant. (* $p < 0.05$, SEM, all experiments were repeated at least 3 times).

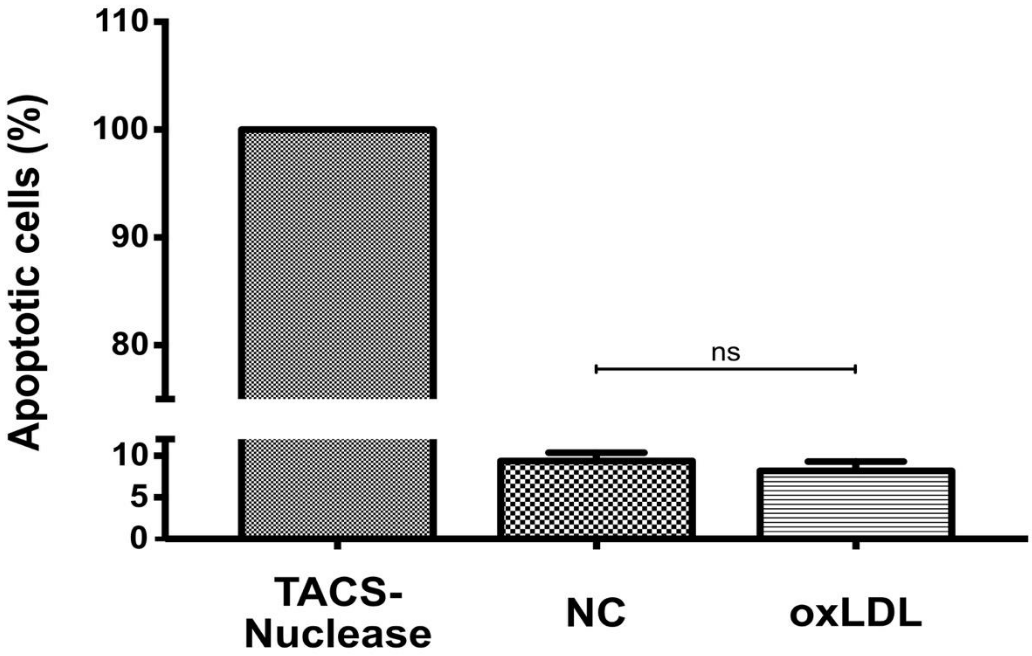

Figures S3

Low dose oxLDL treatment does not induce apoptosis in SMCs. Cells were treated with 10 µg/ml oxLDL for 24h or were left untreated (NC). On day 5 apoptosis was analyzed. TACS-Nuclease treated cells served as a positive control. (* $p < 0.05$, SEM, all experiments were repeated at least 3 times).

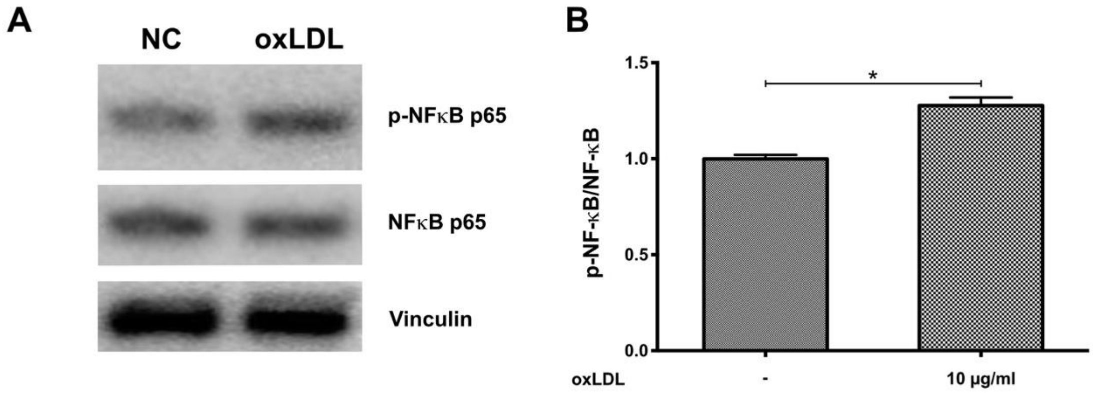

Figure S4

OxLDL priming induces phosphorylation of NFκB p65. Cells were treated with 10 µg/ml oxLDL for 4h and cell lysates were analyzed by western blot. (* $p < 0.05$, SEM, all experiments were repeated at least 3 times).

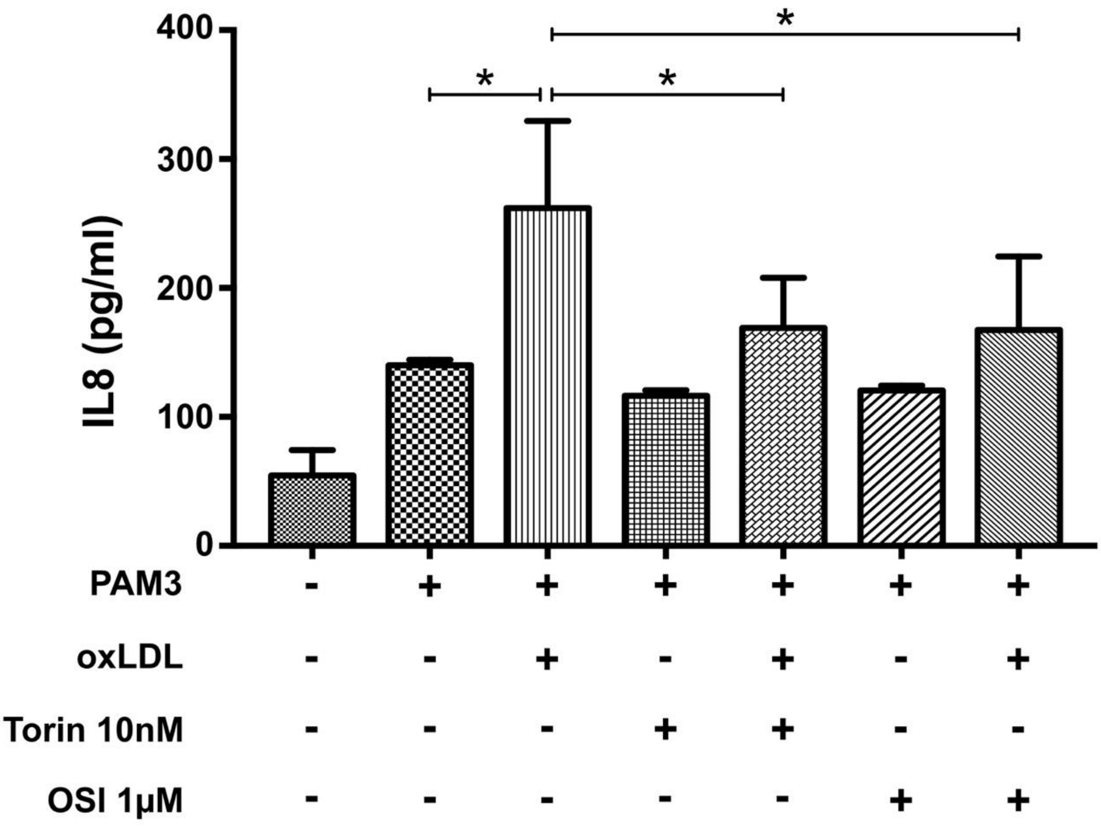

Figure S5

Inhibition of mTOR-signaling pathway blocks oxLDL Priming. SMCs were pretreated with 10 nM of the mTOR-inhibitor Torin1 or 1 µM of the mTOR-inhibitor OSI27 for 30 min, followed by 10 µg/ml oxLDL for 24h. Cells were restimulated on day 5 with 5 µg/ml PAM3cys4 for 24h and IL8 levels were analyzed in the supernatant. (* $p < 0.05$, SEM, all experiments were repeated at least 3 times).

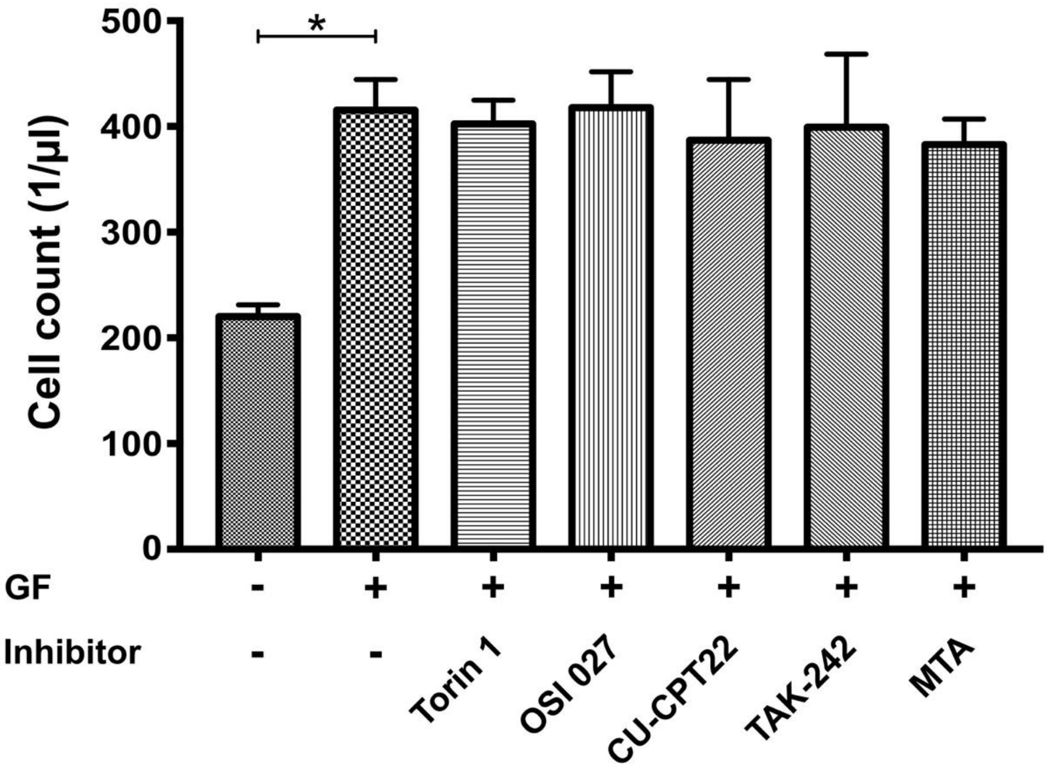

Figure S6

Proliferation of SMCs was not affected by the chemical inhibitors used in this study. Cells were treated with 10 nM of the mTOR-Inhibitor Torin1, 1 µM of the mTOR Inhibitor OSI27, 5 µM of the TLR2-inhibitor CU-CPT22, 1 µM of the TLR4-inhibitor TAK242 or 10 µM of the methyltransferase inhibitor MTA for 24h. On day 5 medium was changed to growth factors supplemented medium (GF), to stimulate proliferation. After 24h cell numbers were counted.

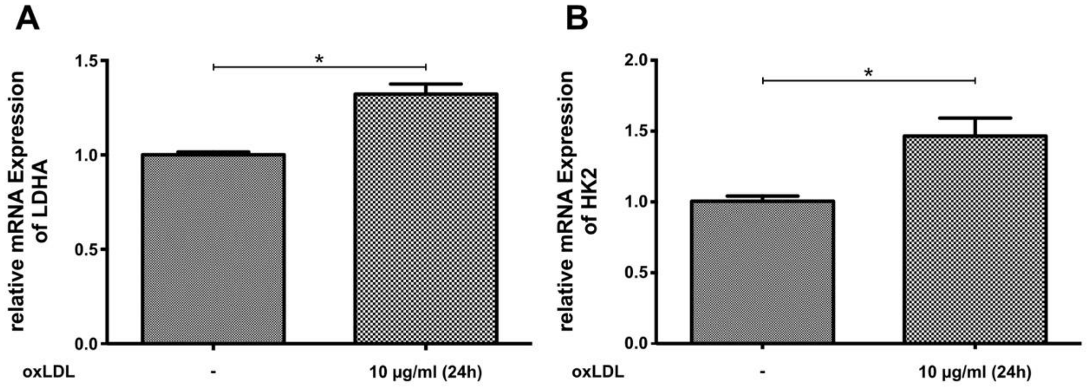

Figure S7

OxLDL priming leads to increased expression of HIF1α target genes. SMCs were treated with 10 µg/ml oxLDL for 12h. mRNA levels of LDHA (A) and HK2 (B) were analyzed by real-time PCR. (* $p < 0.05$, SEM, all experiments were repeated at least 3 times).

Real-time qPCR primer sequences

**Gene**	**Forward primer**	**Reverse primer**
hCD68	GCTACATGGCGGTGGAGTACAA	ATGATGAGAGGCAGCAAGATGG
hMAC2	GGCCACTGATTGTGCCTTAT	AAGCGTGGGTTAAAGTGGAAG
hIL6	AGTGCCTCT TTGCTGCTTTCAC	TGACAAACAAATTCGGTACATCCT
hIL8	ACTGAGAGTGATTGAGAGTGGAC	AACCCTCTGCACCCAGTTTTC
hMCP1	GTGAGGAACAAGCCAGAGCTG	TGCGCAGAATGAGATGAGTTG
hαSMA	AGCAGCTCCAGCTATGTGTGAAGAAG	TTTGTCCCATTCCCACCATCACCC
TFIIB	TCGCCACATTCGCTTCCTGCTTTC	ATATCACCGGCTCTGTAGTCCTCCAC
HSM22α	GCAGATCATCAGTTAGAGCGGAGAGG	AGTTACCATTGCTCAGTGACAGAGCC
LDHA	ATCTTGACCTACGTGGCTTGGA	CCATACAGGCACACTGGAATCTC
HK2	TTGACCAGGAGATTGACATGGG	CAACCGCATCAGGACCTCA
